# Investigating the Residual Effects of Chronic Cannabis Use and Abstinence on Verbal and Visuospatial Learning

**DOI:** 10.3389/fpsyt.2021.663701

**Published:** 2021-06-17

**Authors:** Valentina Lorenzetti, Michael Takagi, Yvonne van Dalen, Murat Yücel, Nadia Solowij

**Affiliations:** ^1^Neuroscience of Addiction and Mental Health Program, Faculty of Health Sciences, Healthy Brain and Mind Research Centre, School of Behavioural and Health Sciences, Australian Catholic University, Melbourne, VIC, Australia; ^2^Child Neuropsychology Unit, Murdoch Children's Research Institute, Melbourne, VIC, Australia; ^3^Faculty of Science, University of Amsterdam, Amsterdam, Netherlands; ^4^BrainPark, School of Psychological Sciences and Monash Biomedical Imaging Facility, The Turner Institute for Brain and Mental Health, Monash University, Melbourne, VIC, Australia; ^5^School of Psychology and Illawarra Health and Medical Research Institute, University of Wollongong, Wollongong, NSW, Australia

**Keywords:** cannabis (marijuana), verbal learning and memory, visuospatial learning, abstinence, tetrahydrocannabinol, California Verbal Learning Test—Second Edition, Brown learning test

## Abstract

**Rationale:** Regular cannabis users have been shown to differ from non-using controls in learning performance. It is unclear if these differences are specific to distinct domains of learning (verbal, visuospatial), exacerbate with extent of cannabis exposure and dissipate with sustained abstinence.

**Objective:** This study examines different domains of learning (verbal, visuospatial) in current and abstaining cannabis users, and the role of chronicity of use.

**Methods:** In a cross-sectional design, we examined 127 psychiatrically healthy participants (65 female) with mean aged of 34 years. Of these, 69 individuals were current regular cannabis users (mean 15 years use), 12 were former cannabis users abstinent for ~2.5 yrs (after a mean of 16 years use), and 46 were non-cannabis using controls. Groups were compared on verbal learning performance assessed via the California Verbal Learning Test (CVLT-II) and for visuospatial learning measured with the Brown Location Test (BLT). We explored the association between CVLT/BLT performance and cannabis use levels in current and former users.

**Results:** Current cannabis use compared to non-use was associated with worse performance on select aspects of verbal learning (*Long Delay Cued Recall*) and of visuospatial learning (*Retroactive Interference* and *LD Rotated Recall*). Prolonged abstinence was associated with altered verbal learning but intact visuospatial learning. There were non-significant correlations between distinct cannabis use measures, age and learning in both current and former users.

**Conclusions:** Our findings suggest cannabis use status (current use, former use) affects different domains of learning (verbal and visuospatial) in a distinct fashion. These findings might be accounted for in the design of cognitive interventions aimed to support abstinence in cannabis users.

## Introduction

Cannabis is the most widely used illicit substance globally (1) and its potency has doubled over the past decade (2, 3). These statistics are concerning as a substantial proportion of cannabis users consume it on a regular basis (4, 5) and a significant minority of people with regular use experience lower school attainment, depression, anxiety, psychosis, impulse-control disorders, suicidal ideation and addiction (6). While there is much to learn about the cognitive correlates of regular cannabis use, a growing body of research has produced increasing knowledge in this area.

One of the core features of regular cannabis use entails alteration of cognitive performance that last beyond acute intoxication, which is thought to reflect chronic residual effects of regular cannabis use (7–12). In particular, altered cognitive function in regular cannabis users affects the domains of learning and memory, which are critical for performing daily tasks, at school and at work (7–13). Therefore, learning and memory alterations in regular cannabis users may underscore lower academic attainment (14, 15) and occupational performance found in cannabis using samples (16, 17).

Poorer learning and memory performance have been documented in regular cannabis users in both verbal and visuospatial domains. Specifically, relative to controls, regular cannabis users have shown lower verbal learning and recall of words (7–12), lower recall and accuracy of visuospatial performance in a checkerboard test (18), and lower retrieval in the virtual Morris water maze task (19). There are some inconsistencies however, as some of the examined samples have not shown verbal/visuospatial learning and memory alterations, or alterations with small to moderate effect sizes (10–12, 18–22). Replication studies in larger samples are required to validate and further examine the association between cannabis use and learning/memory performance, particularly for the visuospatial domain which has been examined by few studies to date.

The role of the extent of cannabis exposure on verbal/visuospatial learning and memory alterations is unclear. Evidence suggests that higher chronicity of use predicts worse verbal learning and memory performance (23–29). However, there is a lack of empirical studies that tested in detail how distinct measures of cannabis exposure affect verbal/visuospatial learning. Such measures include: dosage, duration, age of use onset, hours from last use, potency (9, 10, 12, 30) and cannabinoids such as the main psychoactive compound Δ^9^-tetrahydrocannabinol (THC) that determines cannabis potency (31–33). The lack of studies on how cannabis use patterns and THC levels affect learning performance in chronic users, creates a knowledge gap to inform users, educators, clinicians and policy makers about which measures and levels of cannabis exposure may be more harmful for verbal/visuospatial learning in chronic cannabis users.

Another issue yet to be elucidated is whether poorer verbal/visuospatial learning performance in regular cannabis users persists beyond prolonged abstinence, and the relevant evidence to date is mixed. Some studies show that learning deficits persist beyond abstinence [i.e., after 1 month (17, 30)]. Other studies found that learning alterations are attenuated (e.g., lower effect size) with longer abstinence (12) [e.g., over 4 to 8 weeks (34). Other studies show attenuated learning deficits in cannabis users who abstain for a variety of periods (11): 3 weeks (35), 1 month (36–40), 3 months (41)]; 12 months (35, 42–46). The inconsistency between study findings may be due to methodological confounds, such as long-lasting residual effects of chronic exposure e.g., cumulative lifetime exposure prior to quitting (30).

In sum, lower verbal learning performance has been (largely) consistently identified in chronic, long-term cannabis users. However, visuospatial learning deficits are largely unexplored, as well as the role of cannabis exposure levels and of prolonged abstinence.

The primary aim of this study was to address this evidence gap and to examine whether current cannabis use is associated with selective impairment of either verbal or visuospatial learning and memory. We also aimed to explore the role of extent of cannabis exposure and of prolonged abstinence on verbal/visuospatial learning and memory in chronic cannabis users.

To do this, we recruited 127 people (65 females), consisting of 69 current users and 12 former cannabis users and 46 non-using controls, comprehensively characterized for extent of substance use (alcohol, tobacco and other illicit drug use; cannabis use frequency, quantity, duration, age of onset, time from last cannabis use) and mental health (anxiety, depression and psychotic symptoms). Based on the existing evidence, we hypothesized that worse verbal and visuospatial learning performance would be apparent in cannabis users with more chronic levels of exposure.

## Materials and Methods

### Participants

We recruited 127 people aged between 18 and 55 years via advertisements in local newspapers and Internet websites, and screened using a structured telephone interview to determine study eligibility. Participants included 69 current chronic regular cannabis users, 12 former chronic cannabis users and 46 non-using controls (henceforth called “current users,” “former users,” and “controls,” respectively). All groups were age matched.

### Inclusion and Exclusion Criteria

Cannabis users were included if they: (i) used cannabis at least twice a month for >2 years (the vast majority were currently using >3 days a week over many years, with a median of 30 smoking days/month and of 13 years of regular use); (ii) refrained from using substances other than cannabis, alcohol and tobacco in the month prior to assessment. Exclusion criteria for all participants were: (i) neurological disorders or serious head injury; (ii) Intelligence Quotient (IQ) <70; (iii) current regular use of illicit substances other than cannabis (amphetamines, benzodiazepines, cocaine, ecstasy, hallucinogens, inhalants and opiates; median lifetime use was between 0 and 6 occasions for any other drug).

All participants were requested to abstain from cannabis for at least 12 h prior to testing to enable examining cognitive function in a non-intoxicated state, and provided written informed consent in accordance with local ethics committee guidelines. Ethics approval was given by the Mental Health Research and Ethics Committee (MHREC, I.D. number 459111), the Melbourne Health and North Western Mental Health (Melbourne, Australia), the Wollongong Human Research Ethics Committee (HREC, project number NSA07/03), and the ethics committee of the Murdoch Children's Research Institute (MCRI).

### Procedure

Participants underwent a comprehensive 2.5 h long assessment of mental health, substance use and cognitive function.

#### Mental Health

We screened for psychiatric disorders through the Structured Clinical Interview for the Diagnostic and Statistical Manual for Mental Disorders IV-R (47), and assessed global functioning via the Global Assessment of Functioning module of the DSM-IV (48). We examined psychopathology symptoms of anxiety (State and Trait Anxiety Inventory, STAI; (49), depression and psychosis (Community Assessment of Psychic Experiences, CAPE (50).

#### Substance Use

We assessed lifetime and past month substance use through semi-structured interviews, including the Substance Use History [Orygen Youth Health Research Centre, Melbourne, Australia (51–53)], a detailed structured assessment interview for cannabis (27), and the Timeline Follow-Back (54). From these interviews, we derived levels of tobacco use (cigarettes per week) and cannabis use (i.e., lifetime and past year cumulative dosages and frequencies of use, duration of use and age of onset).

We converted cannabis dosage to standardized units (i.e., cones, approximately equivalent to ~ 0.1 g) (55). We measured urinary levels of the carboxy metabolite of THC (THC-COOH) via toxicology analysis, and THC accumulated in hair. Alcohol use (standard drinks per month) were quantified from the structured interviews and the Alcohol Use Disorders Identification Test (56).

#### Cognitive Function

We assessed *current IQ*, via the Wechsler Abbreviated Scale of Intelligence (WASI) (57) and *premorbid IQ* using the Wechsler Test of Adult Reading (WTAR) (58), respectively.

We measured *verbal learning and memory* via the California Verbal Learning Test, Second Edition (CVLT-II) (59), which was administered according to the manualized instructions. First, participants were asked to recall a list of 16 words presented orally (*List A*) for five consecutive trials (learning *Trials 1 to 5*). Then, participants were instructed to recall a new list of 16 words (*List B*). Subsequently, participants were asked to recall the words from *List A* without any cues to aid memory, and then with cues (*Short Delay Free Recall* and *Short Delay Cued Recall*). After a 20 min interval, the latter procedure was repeated (*Long Delay Free Recall* and *Long Delay Cued Recall*). Finally, participants were asked if they recognized, from among a list of 48 words including distractors, those words that were previously presented in *List A* (*Recognition Trial*).

*Visuospatial learning* was examined using the Brown Location Test [BLT (60)], a visuospatial analog of the CVLT-type verbal learning tasks. Participants were presented with 12 pages, one at a time, on which 58 identically sized black outlined circles were located. At each presentation, one of the circles was filled with a red dot and the location of the red dot was different on each page, thus forming a “list” of 12 red dot-locations to remember akin to the list of words to be remembered on each trial of a verbal learning task. After each trial (i.e., the serial presentation of the 12 pages), the participant was provided an identical page of circles where none were filled with red, and was asked to place red chips in the locations where the red dots had been presented. This procedure was repeated 5 times (learning *Trials 1 to 5*).

Then, participants were presented with a new series of 12 black dots, which they were also asked to recall as above (*Interference* trial). Subsequently, participants were asked to recall the locations of the red dots from *Trials 1–5* immediately after Interference (*Short Delay Recall*), after 20 min delay (*Long Delay Recall*), and after rotating the recall page 90 degrees. Finally, participants were presented with a page containing the original location of the red dots, and additional distractor red dots. Participants were asked to distinguish known 12 dot locations from 12 distractor dot locations (*Recognition* trial).

### Statistical Analyses

#### Descriptive Demographic, Clinical, and Substance Use Data

As the majority of variables were skewed and not transformable, group comparisons for descriptive purposes were performed using chi-square tests for categorical variables (sex); as well as ANCOVAs for normally distributed discrete variables (IQ, STAI) and Kruskal-Wallis tests, followed by *post hoc* Mann-Whitney U tests, for non-normally distributed discrete variables.

#### Primary Aim

To examine group differences for CVLT and BLT performance, Quade's method (61) was used for not normally distributed data from the CVLT and the BLT. Quade's method enables running non-parametric tests comparing groups using covariates. With Quade's method, the dependent variable in ANOVA is the unstandardized residual of a linear regression between the ranked (in ascending order) dependent variable (CVLT and BLT scores) and the ranked covariates (IQ for CVLT, IQ, and age for BLT).

Comparisons between CVLT and BLT trials *within* groups were performed using Friedman- and *post-hoc* Wilcoxon signed rank tests.

#### Exploratory Correlations

Spearman's correlations were run to investigate how performance on the CVLT and BLT (residualized data after regressing out the effects of IQ on the CVLT data, and that of IQ and age on the BLT data) was associated with (i) THC or THC-COOH in hair and urine, respectively; and (ii) extent of cannabis use [duration, age at use onset, dosage (lifetime cumulative cones), frequency (smoking days/month), and hours since last cannabis use].

For all correlations, we utilized the conservative Bonferroni method to control for multiple tests and therefore readjusted the significance threshold to α = 0.0005.

#### Covariates

We retained IQ as a covariate in all analyses of CVLT and BLT data. We used age as an additional covariate in BLT data analyses, as age was significantly associated with BLT measures in non-users. Sex was used as a within-groups factor in analyses of CVLT but not BLT performance, as it significantly affected the former but not the latter. Alcohol standard drinks/month, tobacco cigarettes/week and sub-diagnostic psychopathology symptoms (i.e., anxiety, depression, positive and negative psychotic symptoms) were not included as covariates because they did not significantly affect CVLT and BLT performance.

#### Sensitivity analyses

A series of two sensitivity analyses were performed to confirm the robustness of the effects. First, all analyses were repeated excluding 12 chronic users who used any illicit substances other than cannabis and these confirmed the results from the analyses run with the whole sample. Therefore, we report the results from the whole group analyses.

Second, we reran group comparisons without 7 current users who on the day of testing admitted to having used cannabis for less than the required at least 12 h—abstinence (range 3–10 h, median of 4 h). As these individuals did not show overt signs of acute intoxication, we proceeded with testing.

All analyses were conducted using SPSS, Version 21 (IBM).

## Results

Table 1 summarizes data on demographic, clinical and substance use measures for chronic users, former users and non-users.

**Table 1:** Socio-demographic, cannabis and other substance use, and psychopathology symptoms current and former chronic cannabis users and controls.

		**Controls**	**Cannabis users**	**Former cannabis users**	**Z**	***p***	**df**
**Socio-demographic, IQ, alcohol/tobacco**							
Total *N* [female]^†^^‡^		46 (26)	69 (37)	12 (2)	χ^2^6.53	0.038	_2_
Age, yrs		31.17 (12.83)	32.68 (11.17)	37.83 (11.03)	3.34	0.188	_126_
Education, yrs^*^		13.97 (1.60)	12.82 (2.25)	13.17 (2.41)	7.90	0.019	_122_
Premorbid IQ		106.78 (10.18)	101.40 (13.29)	106.75 (5.01)	5.55	0.062	_125_
IQ^*^^‡^		111.76 (10.91)	104.15 (10.72)	112.25 (11.21)	7.22	0.001	_125_
Global functioning^*^^†^		85.5 (4.95)	73.7 (9.77)	75.58 (9.64)	50.32	<0.001	_126_
Alcohol use (drinks/mo)^†^^‡^		18.49 (23.89)	24.52 (32.74)	52.48 (32.80)	9.43	0.009	_126_
Tobacco (cigarettes/week)^*^^†^^‡^		4.95 (16.79)	58.95 (53.15)	29.33 (38.16)	56.56	<0.001	_126_
**Cannabis use**							
Frequency, days/mo	Lifetime^‡^	_NA_	23.24 (6.89)	28.83 (4.04)	−3.63	<0.001	_80_
	Past 12 mo^‡^	_NA_	24.59 (8.62)	3.33 (8.51)	−4.81	<0.001	_80_
Dosage, cones	Lifetime	_NA_	69,183 (73,271)	43,036 (36,492)	−1.17	0.241	_80_
	Past 12 mo^‡^	_NA_	5,070 (4039)	136.3 (375.3)	−5.30	<0.001	_80_
Duration of regular use, yrs		_NA_	15.13 (10.00)	15.92 (9.61)	−0.297	0.767	_80_
Onset age, yrs		_NA_	16.96 (3.93)	17.33 (3.75)	−0.510	0.610	_80_
Abstinence duration, yrs		_NA_	_NA_	2.46 (5.56)	_NA_	_NA_	_12_
**Psychopathology symptom scores**							
Trait anxiety, STAI		33.64 (7.39)	41.99 (12.19)	35.33 (13.39)	2.13	0.12	_124_
Depression, CAPE^*^		12.29 (2.69)	15.06 (3.87)	13.58 (2.75)	16.34	<0.001	_125_
Positive psychotic, CAPE^*^		24.56 (3.37)	27.58 (6.21)	25.82 (3.22)	7.46	0.024	_121_
Negative psychotic, CAPE^*^		22.26 (5.11)	26.51 (6.95)	23.42 (13.39)	11.95	0.003	_122_

*NA, not applicable;*

* *Significant difference between cannabis users and controls.*

† *Significant difference between former cannabis users and controls*.

‡ *Significant difference between cannabis users and former cannabis users. χ^2^, for results from Kruskal-Wallis test; Z for results from Mann-Whitney U test; F values for IQ and anxiety. Current and premorbid IQ measured with the Wechsler Abbreviated Scale of Intelligence and the Wechsler Test of Adult Reading, respectively; Alcohol use measured with the Alcohol Use Disorders Identification Test; Anxiety symptoms measured with the State and Trait Anxiety Inventory (STAI), depression, positive and negative symptoms measured with the subscales of the Community Assessment of Psychic Experiences (CAPE); Global functioning measured with the module of the DSM-IV*.

### Socio-Demographic and IQ Data

All groups were matched by age and premorbid IQ. Chronic users and controls had an equal composition of males and females. However, former users had a lower proportion of females to males, relative to both chronic users (χ^2^ = 5.82, *p* = 0.016, respectively) and non-users (χ^2^ = 6.05, *p* = 0.014).

Education years were lower in chronic users than non-users (Z = −2.79, *p* = 0.005), and IQ was also lower in chronic users than the other groups (i.e., controls [t = −3.71, *p* < 0.001], former users [t = −2.42, *p* = 0.018]). Global functioning was lower in both cannabis groups than controls (i.e., chronic users, Z = −6.96, *p* = <0.001; and former users, Z = −3.80, *p* < 0.001).

### Alcohol and tobacco Use level

Alcohol use (standard drinks/month) was greater in former users compared to all groups (i.e., current users: Z = −2.48, *p* = 0.013, and controls: Z = −2.83, *p* = 0.005). Tobacco use (cigarettes/week) was greater in current users than former users, and lowest in controls (Z = −2.13, *p* = 0.034 and Z = −3.24, *p* = 0.001, respectively).

### Cannabis Use level

The cannabis groups had similar cannabis use' duration, age of onset and lifetime dosage (see Table 1). However, current users smoked more days/week and consumed a greater amount of cannabis in the past year than former users.

Abstinence duration in current users was median of 16 h (range 3–336 h). Seven current cannabis users reported abstaining for 3 to 10 h despite our request to abstain for at least 12 h; and therefore analyses were repeated excluding these very recent users. Abstinence duration in former users was a mean of 2.5 years (median 6 months, range 1 month−19 years); 9 former users had ceased cannabis use within the past 12 months.

### Subclinical Psychopathology Symptoms

Symptom severity for anxiety, depression and psychosis was greater in current users than controls (t = 4.11, *p* < 0.001; Z = −4.04, *p* = <0.001; Z = −2.66, *p* = 0.008; Z = −3.41, *p* = 0.001, respectively), but did not differ between the other groups (p = n.s.).

### Group Differences in CVLT Performance

Table 1 Overviews group differences in CVLT performance. Group differences in CVLT trials are overviewed below, followed by learning curves and learning trials in each of the three groups (controls, current users, former users).

### CVLT Trials

Group differences emerged for 7 out of the 18 CVLT variables: *Trials 1, 3, 1–5, B, Short Delay Free Recall, Long Delay Free Recall* and *Long Delay Cued Recall* (see Table 2).

**Table 2:** CVLT-II performance in *current* users, *former* users, and controls: mean (standard deviation).

**CVLT trials**	**Controls**	**Current users**	**Former users**	**F-value**	***p***	**df**
Trial 1^*^^†^	8.30 (2.05)	6.96 (2.29)	6.62 (2.14)	5.09	**0.01**	_127_
Trial 2	11.39 (2.33)	10.30 (2.65)	9.92 (2.14)	1.53	0.22	_127_
**Trial 3^†^**	13.07 (1.93)	11.77 (2.66)	11.00 (2.68)	3.67	**0.03**	_127_
Trial 4	13.59 (2.04)	12.35 (2.51)	12.15 (2.94)	1.95	0.15	_127_
Trial 5	13.91 (1.87)	13.00 (2.51)	12.69 (2.69)	0.91	0.41	_127_
Trials 1–5^*^^†^	60.26 (7.98)	54.38 (10.92)	52.38 (10.56)	3.85	**0.02**	_127_
List B^*^	7.07 (2.02)	5.59 (2.18)	6.00 (2.16)	5.40	**0.01**	_127_
Short delay free recall^*^	12.98 (2.24)	11.42 (2.87)	11.38 (2.99)	2.74	**0.07**	_127_
Short delay cued recall	13.46 (2.00)	11.96 (3.07)	12.00 (2.77)	2.10	0.13	_127_
Long delay free recall^*^	13.65 (2.07)	11.91 (3.11)	11.77 (2.98)	3.65	**0.03**	_127_
**Long delay cued recall^†^**	13.87 (1.89)	12.28 (2.95)	11.77 (3.11)	3.35	**0.04**	_127_
Recognition	15.28 (0.958)	14.67 (1.50)	15.38 (0.961)	2.14	0.12	_127_
Recognition false positives	0.91 (1.75)	1.67 (2.42)	1.92 (2.96)	0.62	0.54	_127_
Repetitions	4.30 (3.81)	4.49 (4.06)	4.46 (5.24)	0.02	0.98	_127_
Intrusions	2.07 (2.99)	2.22 (2.92)	3.77 (5.31)	0.10	0.91	_127_
Proactive interference	1.24 (2.43)	1.36 (2.14)	0.62 (1.50)	1.09	0.34	_127_
Retroactive interference	0.94 (1.78)	1.58 (1.81)	1.31 (1.75)	1.03	0.36	_127_
Loss after consolidation	0.26 (1.73)	1.09 (1.88)	0.92 (11.71)	1.50	0.23	_127_

* *Significant difference between cannabis users and controls*.

† *Significant difference between former cannabis users and controls. CVLT-II, California Verbal Learning Test Version 2. Proactive Interference, prior learning interfering with new learning (Trial 1- List B); Retroactive Interference, later learning interfering with previous learning (Trial 5—Short Delay Free Recall) and Loss after Consolidation, loss of recalled words after delay (Trial 5—Long Delay Free Recall)*.

#### Current Users vs. Controls

Cannabis users performed worse than controls for CVLT *Trial 1* (F = 6.52, *p* = 0.012), and learning from *Sum Trials 1–5* (F = 4.54, *p* = 0.035). Additionally, current users recalled less words than controls for CVLT *List B* (F = 10.08, *p* = 0.002), *Short Delay Free Recall* (F = 4.37, *p* = 0.039) and *Long Delay Free Recall* (F = 5.12, *p* = 0.026). These group differences did not survive a sensitivity analyses that we ran after excluding seven current users who consumed cannabis recently <12 h before testing.

After running the sensitivity analysis, the only performance difference that emerged in cannabis users vs. controls was poorer performance by users in *Long Delay Cued Recall* (F = 4.35, *p* = 0.015).

#### Former Users vs. Controls

Similarly to current users, former users vs. controls showed lower CVLT performance for *Long Delay Cued Recall* (F = 4.42, *p* = 0.040), *Trial 1* (F = 6.95, *p* = 0.011), and learning from *Sum Trials 1–5* (F = 5.14, *p* = 0.027). Additionally, former users performed worse than controls on *Trial 3* (F = 6.04, *p* = 0.017).

### CVLT Learning Curves

**Figure 1:**
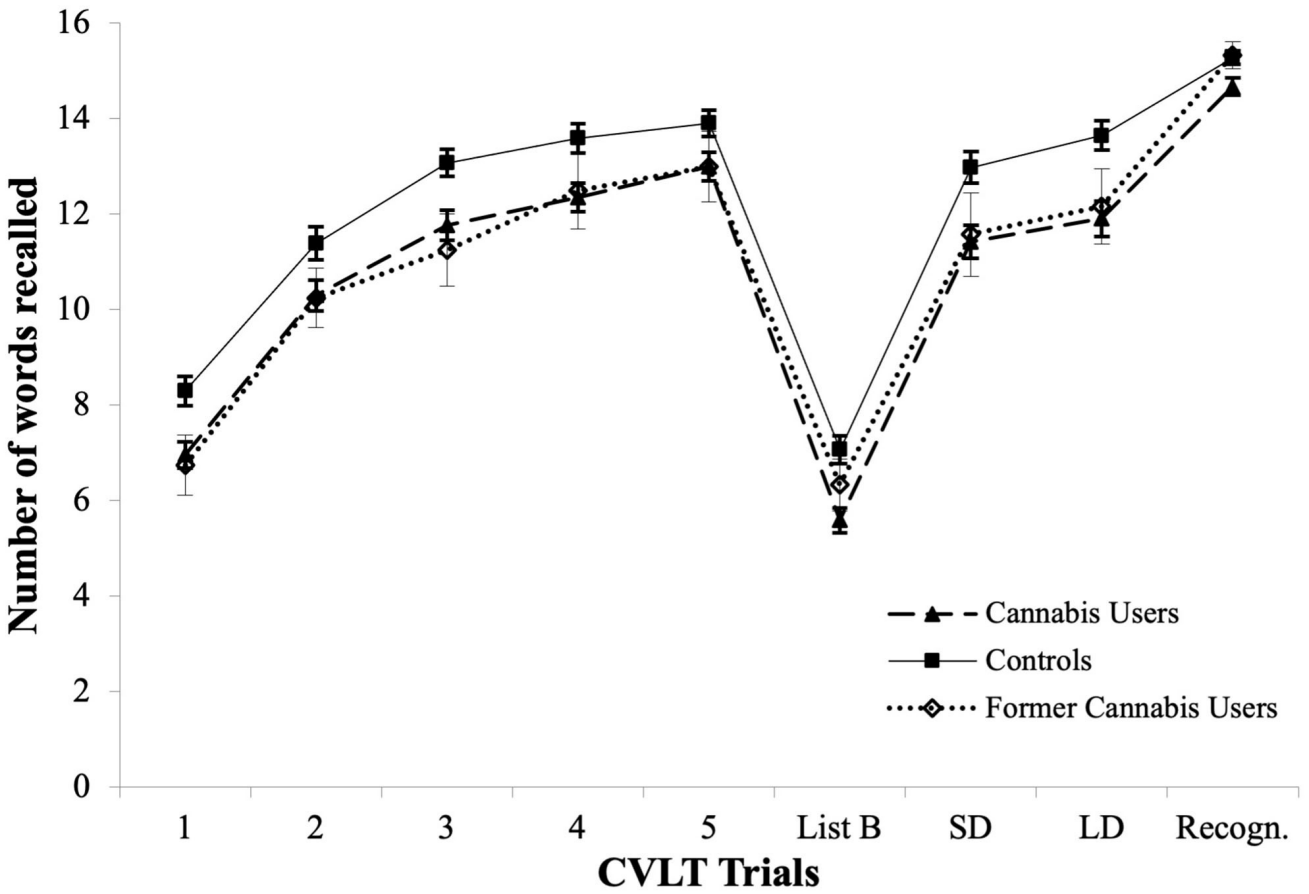
CVLT learning curves across Trials 1–5, in current cannabis users, former cannabis users and controls, and mean performance for List B, Short Delay Free Recall (SD), Long Delay Free Recall (LD), and Recognition Trial (Recogn.).

As shown in Figure 1, CVLT learning curves improved for all groups at every trial from *Trial 1* to *Trial 4* (all *p* < 0.001). Controls improved word recall at every trial from *Trial 1* to *Trial* (Z range from −5.71 to −4.54, *p* < 0.001). Similarly to controls, current users improved word recall at *every trial from Trial 1 to Trial* (Z range from −6.83 to −2.58, *p* < 0.01). Former users only significantly improved their word recall from *Trial 1* to *Trial 2* (Z = −3.08, *p* = 0.002).

### CVLT Delayed Recall

#### Controls

Controls showed a significant increase in the ***Long*** *Delay* ***Free*** *Recall vs*. ***Short*** *Delay Free Recall* (Z = −3.09, *p* = 0.002) and ***Long*** *Delay* ***Cued*** *Recall vs*. ***Short*** *Delay Cued Recall* (Z = −3.25, *p* = 0.001).

Additionally, controls improved significantly only after cues were given, in the *Short Delay Cued Recall* trial (Z = −2.14, *p* = 0.032).

#### Current Users

Similarly to controls, current users showed a significant increase in the ***Free Long*** *Delay Recall* vs. ***Free Short*** *Delay Recall* (Z = −2.79, *p* < 0.005) and ***Cued Long*** *Delay Recall vs*. ***Cued Short*** *Delay Recall* (Z = −2.28, *p* = 0.023). In contrast to controls, current users performed better during *Cued Recall vs. Free Recall* for the *Short Delay* trial (Z = −3.06, *p* = 0.002) and for the *Long Delay* trial (Z = −2.12, *p* = 0.034).

#### Former Users

Former users did not show differences in any delayed recall trials.

### Exploratory Correlations Between CVLT Performance, Cannabis Use Measures, and Age

Older age was not associated with any CVLT variables in cannabis users, but was associated with better recall at *Trial 5* (rs = 0.465, *p* = 0.001) and more *Intrusions* in controls (rs = 0.291, *p* = 0.045) and with greater *Recognition of False Positives* in former users (rs = 0.602, *p* = 0.029).

#### Current Users

Greater cannabis frequency over the lifetime was associated with lower *Retroactive Interference* and greater *Loss After Consolidation*; and greater frequency of cannabis use in the past year was associated with worse recall at *Trial 1*. More cumulative cannabis dosage in the lifetime was correlated with worse recall at *Trial 1* and worse *Retroactive Interference*; and greater past year cumulative dosage was correlated with worse recall at *Trial 1*. Earlier age of onset was correlated with less *Intrusions*, lower *Retroactive Interference* and greater *Proactive Interference*.

#### Former Users

In former users, greater frequency of cannabis use in the past year was associated with greater recall at *Trial 1*, greater *Recognition of False Positives* and lower *Intrusions*. Greater past year cumulative dosage was correlated with worse recall at *Trial 4*. Earlier age of onset was correlated with better recall at *Trials 1, 2, 3, 4, 1–5*, and *Trial B*.

None of the correlations run between CVLT performance and cannabis use levels in current and former users survived Bonferroni correction for multiple tests (see Supplementary Tables 1, 2).

### Group Differences in BLT Performance

#### BLT trials

One cannabis user did not complete the BLT, hence for these analyses *n* = 68 current cannabis users. BLT performance differed between groups for 2 of the 15 variables (see Table 3). Current users performed significantly worse than controls on *Retroactive Interference* and on *Long Delay Rotated Recall* (F = 5.95, *p* = 0.016 and F = 1.62, *p* = 0.014). Current users performed worse than former users on *Short Delay Free Recall* (F = 4.28, *p* = 0.042).

**Table 3:** BLT performance in *current* cannabis users, *former* cannabis users and *controls*: mean (standard deviation).

**BLT trials**	**Controls**	**Current users**	**Former users**	**F**	***p***	**_**df**_**
Trial 1	3.93 (2.03)	3.93 (2.01)	3.54 (1.56)	0.05	0.95	_126_
Trial 2	5.85 (2.38)	5.24 (2.10)	5.85 (1.95)	0.47	0.62	_126_
Trial 3	7.00 (2.69)	6.22 (2.44)	6.46 (2.79)	0.54	0.58	_126_
Trial 4	8.72 (2.66)	7.26 (2.69)	7.85 (3.39)	1.80	0.17	_126_
Trial 5	8.72 (2.86)	7.78 (2.92)	8.77 (2.92)	0.82	0.45	_126_
Trials 1–5	34.22 (10.84)	10.43 (9.90)	32.46 (9.17)	0.73	0.49	_126_
Interference	3.72 (2.04)	3.19 (1.72)	3.23 (1.42)	0.04	0.96	_126_
**Short delay free recall^‡^**	7.93 (2.93)	6.22 (2.92)	8.15 (3.13)	3.74	**0.03**	_126_
Long delay free recall	7.76 (2.81)	6.50 (2.87)	7.23 (3.68)	1.40	0.25	_126_
Long delay rotated recall^*^	7.15 (3.18)	5.34 (2.87)	6.69 (3.43)	2.53	**0.08**	_126_
Recognition	18.70 (3.41)	16.78 (3.46)	17.69 (4.68)	1.80	0.17	_126_
Recognition false positives	3.00 (2.29)	3.99 (2.28)	3.00 (2.31)	1.60	0.21	_126_
Proactive interference	0.22 (2.30)	0.74 (2.35)	0.31 (2.53)	0.19	0.83	_126_
Retroactive interference^*^	0.78 (1.53)	1.56 (2.10)	0.62 (1.56)	3.51	**0.03**	_126_
Loss after consolidation	0.96 (1.66)	1.28 (1.91)	1.54 (1.51)	1.15	0.32	_126_

* *Significant difference between cannabis users and controls*.

‡ *Significant difference between cannabis users and former cannabis users. Proactive Interference, prior learning interfering with new learning (Trial 1—List B); Retroactive Interference, later learning interfering with previous learning (Trial 5—Short Delay Free Recall) and Loss after Consolidation, loss of recalled words after delay (Trial 5—Long Delay Free Recall)*.

##### Sensitivity analyses for BLT trials

After exclusion of seven participants who reported using cannabis within 12 h of the assessment, impaired performance persisted in current users vs. controls, for both *Retroactive Interference* (F = 3.29, *p* = 0.041) and *Long Delay Rotated Recall* (F = 3.13, *p* = 0.048).

#### BLT learning curves

**Figure 2:**
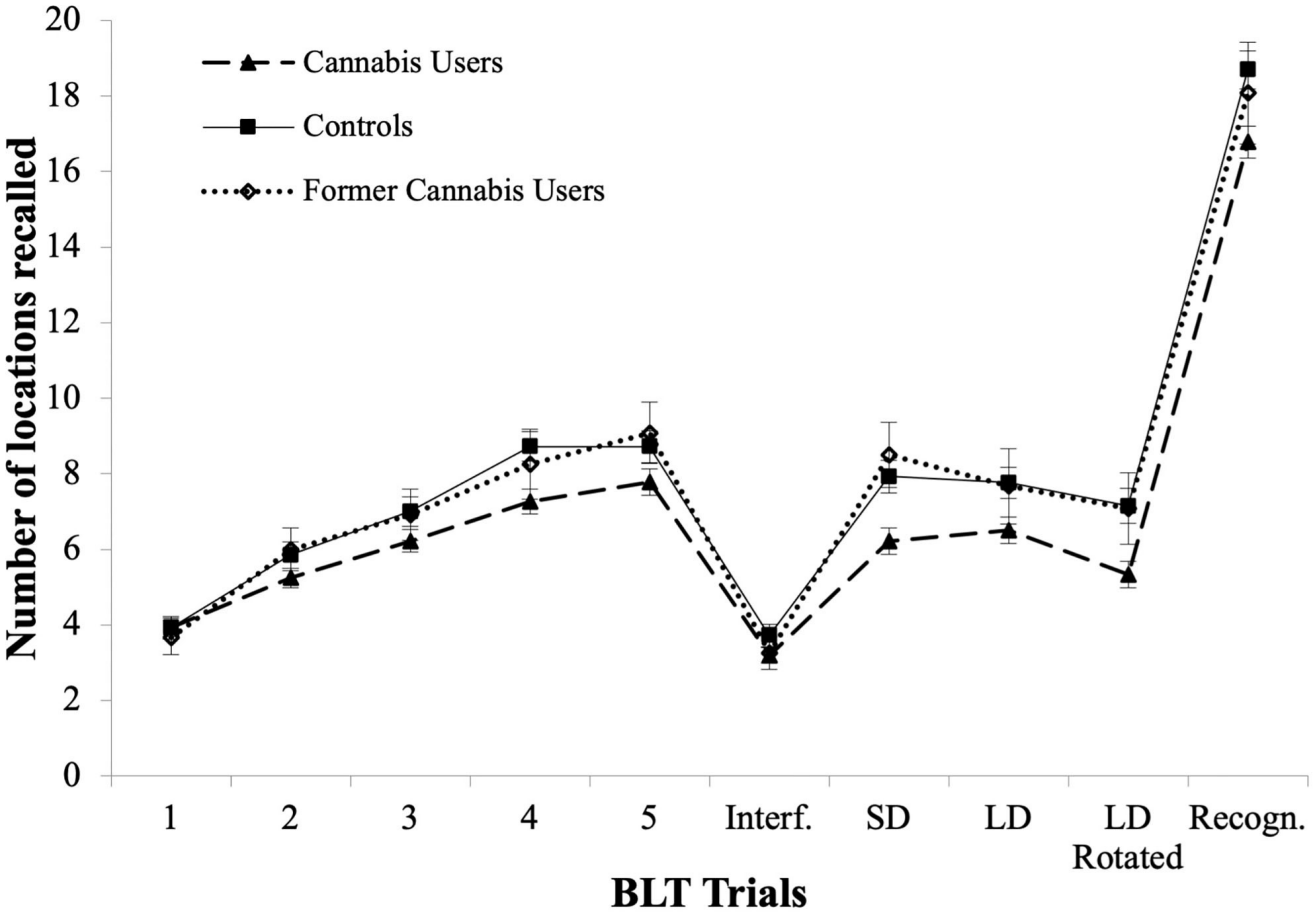
BLT learning curves for Trials 1–5, in current users, former cannabis users and controls, as well as for Interference Trial (Interf.), Short Delay Free Recall (SD), Long Delay Free Recall (LD), Rotated Long Delay Recall (Rotated), and Recognition Trial (Recogn.).

BLT learning curves are shown in Figure 2. The pattern of results was identical to that of the CVLT: current users, former users and controls showed significant improvement in recall [$\chi_{\left(4,68\right)}^{2}$ = 109.0, *p* < 0.001; $\chi_{\left(4,12\right)}^{2}$ = 24.89, *p* < 0.001 and $\chi_{\left(4,46\right)}^{2}$ = 114.7, *p* < 0.001 respectively]. Current users and controls improved at every trial from *Trial 1 to Trial 4* (Z range between −4.38 and −2.01, *p* < 0.04; and Z range between −4.88 and −3.52, *p* < 0.001, respectively). Former cannabis users only improved between *Trial 1* and *Trial 2* (see learning curves in Figure 2, Z = −2.57, *p* = 0.010).

#### BLT Delayed Recall

Current users showed worse BLT performance in *Delayed Recall* only after *Page Rotation* (Z = −4.76, *p* < 0.001). None of the groups showed a difference between *Short Delay Recall* and *Long Delay Recall*.

#### Exploratory correlations Between BLT Performance, Age, and Cannabis Use Measures

There was no association between age and BLT performance in neither current users, former users and controls.

##### Current users

In current cannabis users, greater lifetime cannabis use frequency was correlated with lower BLT recall at *Trial 1–5 Total*, and greater *Recognition of False Positives*. Greater THC-COOH in urine was associated with lower performance during *Interference* and better performance during the *Proactive Interference* trial.

##### Former users

In former cannabis users, greater frequency of cannabis use in the lifetime was associated with greater recall at BLT *Trial 3, Trial 4, Trial 1–5 Total, Long Delay Free Recall, Long Delay Rotated Recall* and greater *Recognition Hits*. Later age of cannabis use onset was associated with lower recall at *Trial 3* and *Trial 4*, and lower *Recognition Hits*. Greater abstinence duration was associated with greater *Long Delay Free Recall* and *Long Delay Rotated Recall*.

None of the correlations between BLT performance, cannabis use levels and age in any groups survived Bonferroni correction for multiple tests (Supplementary Tables 3, 4).

## Discussion

This study shows that cannabis use status (current vs. former use) and extent of cannabinoid exposure have a differential impact on specific measures of verbal and visuospatial learning. Specifically, select measures of verbal learning in current and former cannabis users were impaired i.e., *Long Delay Cued Recall* of the CVLT. Instead, visuospatial learning was impaired only in current users i.e., *Retroactive Interference* and *Long Delay Rotated Recall* of the BLT. Performance on the CVLT and BLT was not significantly associated with any measures of cannabis exposure.

Poorer CVLT *Long Delay Cued Recall* performance in current cannabis users was robust to sensitivity analyses which excluded 7 people who did not appear intoxicated but admitted having smoked cannabis recently i.e., <12 h before testing (instead of the required >12 h), and persisted in former users. Therefore, lower *Long Delay Cued Recall* may reflect the residual effects of chronic cannabis use that are long-lasting and detectable well-beyond prolonged abstinence. This interpretation is consistent with findings that verbal learning alterations in cannabis users are apparent after THC metabolites are no longer detected in urine (9), and in cannabis users with chronic exposure reflected by long duration [i.e., 16 yrs (25) and 23 yrs (24)], dependent and almost daily use (62).

However, these notions are not supported by the lack of robust correlations between *Long Delay Cued Recall* and any measure of cannabis exposure including abstinence durations and chronicity of use. Therefore, the specific indices of cannabis exposure driving CVLT alterations in these groups of current and former users are unclear. Of note, verbal learning is ascribed by the function of posterior and frontoparietal cortices (41, 46, 63–65) that are high in cannabinoid receptors and thus might be vulnerable to the long lasting effects of repeated impact of cannabinoid exposure via complex neural mechanisms (66).

Surprisingly, most of the CVLT performance differences between current users and controls (i.e., *Trial 1, Sum Trials 1–5, List B, Short Delay Free Recall, Long Delay Free Recall*) were no longer detectable after sensitivity analyses without 7 people who recently smoked cannabis i.e., <12 h before testing. Therefore, recent cannabis exposure may drive alterations of select components of verbal learning alterations (17, 24, 27, 41, 46, 62). This notion is supported by other study findings that better Ray Auditory Verbal Learning Test performance is associated with longer abstinence duration (67) (*List B* (27), *total words recalled* (12, 24). However, we found no significant correlation between better CVLT performance and number of hours from last cannabis use or urinary THC metabolites. Thus, the strength of this finding needs to be verified in future studies that carefully measure abstinence duration (68), as only a few studies of verbal learning to date report how long people abstained from cannabis before testing (25, 35).

Poorer visuospatial learning performance in cannabis users emerged in select BLT measures (*Retroactive Interference* and *Long Delay Rotated Recall*). These differences were robust and survived sensitivity analyses without a subgroup of recent users. Our findings are consistent with reports that visuospatial learning alterations in cannabis users affect *recall* but not *acquisition* trials (19, 45), but contrast previous meta-analytic findings that failed to find group differences (12).

The discrepancy between our results and those from previous work, might be due to systematic differences between sample characteristics and the tools used to assess visuospatial learning. First, our sample was older (i.e., ~34 years) than the meta-analyzed samples i.e., <26 yrs (12). Interestingly, previous evidence shows that aging affects visuospatial learning (69–71). Therefore, lower visuospatial *recall* may be due to altered aging processes in cannabis users. However, the lack of significant correlations between age and BLT performance in this study does not support this notion and is to be further tested in future work. Second, the meta-analyzed studies to date used measures of visuospatial learning other than the BLT. Such measures (e.g., accuracy and total scores from the Rey-Osterrieth and Bender Visual-Motor Gestalt Test) might not have been sensitive enough to detect alterations specific to *recall* rather than *acquisition* trials (12).

The mechanisms underlying altered BLT in *recall* trials in current users are unclear. One candidate mechanism is impaired executive functioning (45, 72–74). Indeed, recall—but not acquisition—relies on executive function (75). Also, aging significantly affects the integrity of para-hippocampal and cingulate cortices that are concurrently ascribed to visuospatial learning, and to the residual effects of regular cannabis exposure (19).

Interestingly, we found for the first time that both current and former users showed similar *Learning Curves* across the verbal and visuospatial domains, suggesting the employment of common learning strategies across both domains (73). This similarity was apparent, given the similar structure of the BLT and the CVLT (i.e., both tests start with 5 *Learning Trials*, followed by an *Interference* trial and *Delayed Recall* trials). The overlapping *learning curves* between current and former users (see Figure 1), suggest that alteration of *learning curves* commences during regular use and does not recover after prolonged abstinence.

There are several limitations to this study. First, we examined a small sample of former users, and our findings require validation in larger samples. Second, our group of current users were *not* matched to controls for level of education, IQ and severity of sub-clinical psychopathology symptoms. Nevertheless, premorbid IQ was matched between groups, and we controlled for current IQ i.e., by using IQ as a covariate in all group comparisons (years of education were not associated with any performance measures and were thus not included as a covariate).

We also minimized potential confounding impact of severe mental health conditions on cognitive performance, by screening for any diagnoses of psychopathology. However, we cannot rule out that sub-clinical psychopathology symptoms in our sample, which are shown to exacerbate cognitive deficits (15), may have driven our findings on cognitive alterations. Since sub-clinical psychopathology symptoms as measured in this study did not exert a significant effect on performance, they were therefore not included as covariates in the analyses. On the other hand, our sample is representative of cannabis users within the general community, where higher (although not diagnosable) psychopathology symptoms and worse cognitive outcomes have been consistently reported (14, 76–79).

Third, correlational analyses were run in current users and former users including males and females, and not in different sexes separately, therefore precluding a detailed understanding of possible sex differences in the emerging alterations. Our strategy mitigated type-1 errors, as we have run a substantial number of correlational analyses in current and former cannabis users across all cognitive variables examined. Fourth, the number of abstinent users (*n* = 12) was small for statistical analyses, and findings pertaining to this group require replication in larger samples. Further, the cross-sectional study design and the lack of information on the sample' resilience levels, prevented to determine if abstinence was the reason for better performance compared to current usage, or the consequence of a latent factor such as resilience that also leads to better performance.

Our findings might be accounted for in the design of cognitive interventions aimed to support abstinence in cannabis users. For example, if a clinical practitioner knew that a patient uses cannabis regularly, and that regular cannabis use is associated with impaired *Long Delay Cued Recall*, the practitioner may implement strategies to ensure that their client recalls information/instructions critical for engaging with the treatment (e.g., repeating or asking the client to repeat the instruction/information, sharing written instructions). Also, knowing that regular cannabis users have worse performance on *Retroactive Interference and LD Rotate Recall*, could indicate to a practitioner that visuospatial information relevant for the treatment (e.g., the location of appointment/testing/treatment sites) may not be retained when learning occurs; and may prompt the practitioner to implement strategies to boost recall of visuospatial information relevant for the patient to attend treatment (e.g., sharing a map or sending a text reminder with information on appointment/testing/treatment sites).

In conclusion, our findings suggest that current cannabis use is associated with chronic residual effects on select aspects of verbal learning (*Long Delay Cued Recall*) and of visuospatial learning (*Retroactive Interference* and *Long Delay Rotated Recall*). Prolonged abstinence in former users was associated with altered verbal learning but intact visuospatial learning, suggesting that abstinence has a different impact on distinct domains of learning. Our findings warrant the conduct of future work that systematically tracks how learning performance in chronic cannabis users is affected during intoxication to map acute-on-chronic effects, residual effects beyond intoxication, and those that remain with prolonged abstinence, to track how the residual-on-chronic effects dissipate over time. Additional studies with careful assessment of cannabis use indices and exposure to THC and other cannabinoids, should examine how these findings extend to cannabis user groups who experience worse mental health outcomes.

## Data Availability Statement

The raw data supporting the conclusions of this article will be made available by the authors, without undue reservation.

## Ethics Statement

Ethics approval was given by the Mental Health Research and Ethics Committee (MHREC, I.D. number 459111), the Melbourne Health and North Western Mental Health (Melbourne, Australia), the Wollongong Human Research Ethics Committee (HREC, project number NSA07/03), and the ethics committee of the Murdoch Children's Research Institute (MCRI). The patients/participants provided their written informed consent to participate in this study.

## Author Contributions

MT and VL drove data collection. VL drafted and revised the manuscript writing and statistical analyses and interpretation. YD contributed to the drafting and the running of the statistical analyses. MY and NS drove the study design. All authors revised the manuscript and contributed to the interpretation of the findings.

## Conflict of Interest

The authors declare that the research was conducted in the absence of any commercial or financial relationships that could be construed as a potential conflict of interest.
