# Supplementary material for: Investigating the Residual Effects of Chronic Cannabis Use and Abstinence on Verbal and Visuospatial Learning
Source: Front Psychiatry. 2021 Jun 17;12:663701. doi: 10.3389/fpsyt.2021.663701 (PMC8247947; doi:10.3389/fpsyt.2021.663701)
Supplement: Supplementary file 1 [file Data_Sheet_1.docx]

**Supplementary materials**

**Supplementary Table 1.** **Correlations between *CVLT* performance (residualised after controlling for IQ) and cannabis use measures in *current* cannabis users: *rho* values.**

| **CVLT** | Lifetime Frequency | Past Year Frequency | Lifetime Dosage | Past Year Dosage | Duration of Use | Age of Onset | Hours since last use | Urine THC-COOH |
| --- | --- | --- | --- | --- | --- | --- | --- | --- |
| **Trial 1** | n.s. | **rs = -.272, p=.025** | **rs = -.287, p=.018** | **rs = -.295, p=.014** | n.s. | n.s. | **rs = .304, p=009** | **rs = -.396, p=.007** |
| **Trial 2** | n.s. | n.s. | n.s. | *rs = -.229, p=.061* | n.s. | n.s. | n.s. | **rs = -.360, p=.015** |
| Trial 3 | n.s. | n.s. | *rs = -.231,* p=.058 | n.s. | n.s. | n.s. | n.s. | n.s. |
| **Trial 4** | n.s. | n.s. | n.s. | n.s. | n.s. | n.s. | n.s. | **rs = -.347, p=.020** |
| Trial 5 | n.s. | n.s. | *rs = -.217, p=.075* | n.s. | n.s. | n.s. | n.s. | n.s. |
| **Trials 1 – 5** | n.s. | n.s. | *rs = -.234, p=.054* | n.s. | n.s. | n.s. | n.s. | **rs = -.299, p=.046** |
| Trial B | n.s. | n.s. | n.s. | n.s. | n.s. | n.s. | *rs = .214, p=071* | .24 |
| Short Delay Free Recall | n.s. | n.s. | n.s. | n.s. | n.s. | n.s. | n.s. | .12 |
| Short Delay Cued Recall | n.s. | n.s. | n.s. | n.s. | n.s. | n.s. | n.s. | -.06 |
| Long Delay Free Recall | n.s. | n.s. | n.s. | n.s. | n.s. | n.s. | n.s. | -.01 |
| Long Delay Cued Recall | n.s. | n.s. | n.s. | n.s. | n.s. | n.s. | n.s. | -.06 |
| Recognition | n.s. | n.s. | n.s. | n.s. | n.s. | n.s. | n.s. | -.06 |
| Recognition False Positives | n.s. | n.s. | n.s. | n.s. | n.s. | *rs = .094, R=.205* | n.s. | -.17 |
| **Repetitions** | n.s. | n.s. | n.s. | n.s. | n.s. | n.s. | *rs = .198, p=095* | **rs = -.366, p=.014** |
| **Intrusions** | n.s. | n.s. | n.s. | n.s. | n.s. | **rs = .265, p==.029** | .11 | -.17 |
| **Proactive Interference** | n.s. | n.s. | n.s. | n.s. | n.s. | **rs = -.277, p=.022** | .10 | ***rs*** *= -.272, p=.070* |
| **Retroactive Interference** | **rs = -.247, p=.042** | n.s. | **rs = -.296, p=.014** | n.s. | n.s. | **rs = .250, p=.040** | .09 | ***rs = -.272, p=.070*** |
| **Loss after consolidation** | **rs = .242, p=.047** | n.s. | n.s. | n.s. | n.s. | n.s. | -.08 | n.s. |

No correlation survived Bonferroni’s correction for multiple comparison.

**Supplementary Table 2.** **Correlations between *CVLT* performance (residualised after controlling for IQ) and cannabis use measures in *former* cannabis users: *rho* values.**

| **CVLT** | Lifetime Frequency | Past Years Frequency | Lifetime Dosage | Past Years Dosage | Duration of Use | Age of Onset | Duration of Abstinence |
| --- | --- | --- | --- | --- | --- | --- | --- |
| **Trial 1** | n.s. | n.s. | n.s. | n.s. | n.s. | **rs = -.700, p=.011** | n.s. |
| **Trial 2** | n.s. | **rs= .616 p=.033** | n.s. | n.s. | n.s. | **rs = -.742, p=.006** | n.s. |
| **Trial 3** | n.s. | n.s. | n.s. | n.s. | n.s. | **rs = -.678, p=.015** | n.s. |
| **Trial 4** | n.s. | n.s. | *rs = -.538, p=.071* | n.s. | **rs = -.578, p=.049** | **rs = -.686, p=.014** | n.s. |
| **Trial 5** | n.s. | n.s. | n.s. | n.s. | n.s. | **rs = -.555 p=.061** | n.s. |
| **Trials 1 – 5** | n.s. | n.s. | n.s. | n.s. | n.s. | **rs = -.806, p=.002** | n.s. |
| **Trial B** | n.s. | n.s. | n.s. | n.s. | n.s. | **rs = -.788, p=.002** | n.s. |
| Short Delay Free Recall | n.s. | n.s. | n.s. | n.s. | n.s. | n.s. | n.s. |
| Short Delay Cued Recall | n.s. | n.s. | n.s. | n.s. | n.s. | n.s. | n.s. |
| Long Delay Free Recall | n.s. | n.s. | n.s. | n.s. | n.s. | n.s. | n.s. |
| Long Delay Cued Recall | n.s. | n.s. | n.s. | n.s. | n.s. | n.s. | n.s. |
| Recognition | n.s. | n.s. | n.s. | n.s. | n.s. | n.s. | n.s. |
| **Recognition False Positives** | n.s. | **rs = -.702 p=.011** | n.s. | **rs = -.595, p=.041** | n.s. | *rs = .558, p=.059* | *rs = .546, p=.053* |
| Repetitions | n.s. | n.s. | n.s. | n.s. | n.s. | n.s. | n.s. |
| **Intrusions** | n.s. | **rs = -.585 p=.046** | n.s. | n.s. | n.s. | n.s. | n.s. |
| Proactive Interference | n.s. | n.s. | n.s. | n.s. | n.s. | n.s. | n.s. |
| Retroactive Interference | n.s. | n.s. | n.s. | n.s. | n.s. | n.s. | n.s. |
| Loss after consolidation | n.s. | n.s. | n.s. | n.s. | n.s. | n.s. | n.s. |

No correlation survived Bonferroni’s correction for multiple comparison. p<.05 in **bold**, p<.1 in *italics*.

**Supplementary Table 3.** **Correlations between *BLT* performance (residualised after controlling for IQ and age) and cannabis use measures in *current* cannabis users: *rho* values.**

| **BLT** | **Lifetime Frequency** | **Past Year Frequency** | **Lifetime Dosage** | **Past Year Dosage** | **Duration of Use** | **Age of Onset** | **Hours since last use** | **Urine THC-COOH** |
| --- | --- | --- | --- | --- | --- | --- | --- | --- |
| Trial 1 | n.s. | n.s. | n.s. | n.s. | n.s. | n.s. | n.s. | n.s. |
| Trial 2 | n.s. | n.s. | n.s. | n.s. | n.s. | n.s. | n.s. | n.s. |
| **Trial 3** | n.s. | n.s. | n.s. | n.s. | n.s. | n.s. | n.s. | n.s. |
| **Trial 4** | n.s. | n.s. | n.s. | n.s. | n.s. | n.s. | n.s. | n.s. |
| Trial 5 | n.s. | n.s. | n.s. | n.s. | n.s. | n.s. | n.s. | n.s. |
| **Trial 1-5 Total** | **rs = .243, p=.041** | n.s. | n.s. | n.s. | n.s. | n.s. | n.s. | n.s. |
| **Interference Trial** | n.s. | n.s. | n.s. | n.s. | n.s. | n.s. | n.s. | **rs** *= -.255, p=.094* |
| Short Delay Free Recall | n.s. | n.s. | n.s. | n.s. | n.s. | n.s. | n.s. | n.s. |
| Long Delay Free Recall | n.s. | n.s. | n.s. | n.s. | n.s. | n.s. | n.s. | n.s. |
| Long Delay Rotated Recall | n.s. | n.s. | n.s. | n.s. | n.s. | n.s. | n.s. | n.s. |
| Recognition Hits | n.s. | n.s. | n.s. | n.s. | n.s. | n.s. | n.s. | n.s. |
| **Recognition False Positives** | **rs = .278 p=.019** | n.s. | n.s. | n.s. | n.s. | n.s. | n.s. | n.s. |
| **Proactive Interference** | n.s. | n.s. | n.s. | n.s. | n.s. | n.s. | n.s. | **rs = .354, p=.019** |
| Retroactive Interference* | n.s. | n.s. | n.s. | n.s. | n.s. | n.s. | n.s. | n.s. |
| Loss after Consolidation | n.s. | n.s. | n.s. | n.s. | n.s. | n.s. | n.s. | n.s. |

No correlation survived Bonferroni’s correction for multiple comparison. p<.05 in **bold**, p<.1 in *italics*.

**Supplementary Table 4. Correlations between BLT performance (residualised after controlling for IQ and age) and cannabis use measures in *former* cannabis users: *rho* values.**

| **BLT** | Lifetime Frequency | Past Year Frequency | Lifetime Dosage | Past Year Dosage | Duration of Use | Age of Onset | Duration of Abstinence |
| --- | --- | --- | --- | --- | --- | --- | --- |
|  |  |  |  |  |  |  |  |
| Trial 1 | n.s. | n.s. | n.s. | n.s. | n.s. | n.s. | n.s. |
| Trial 2 | n.s. | n.s. | n.s. | n.s. | n.s. | n.s. | n.s. |
| **Trial 3** | **rs = .620, p=.024** | n.s. | n.s. | n.s. | n.s. | **rs = -.640, p=.018** | n.s. |
| Trial 4 | **rs = .559, p=.047** | n.s. | n.s. | n.s. | n.s. | **rs = -.568, p=.043** | *rs = -.541, p=.056* |
| **Trial 5** | n.s. | n.s. | n.s. | n.s. | n.s. | n.s. | n.s. |
| **Trial 1-5 Total** | **rs = .620, p=.024** | n.s. | n.s. | n.s. | n.s. | n.s. | n.s. |
| Interference Trial | n.s. | n.s. | n.s. | n.s. | n.s. | n.s. | n.s. |
| **Short Delay Free Recall** | n.s. | n.s. | n.s. | n.s. | n.s. | n.s. | n.s. |
| **Long Delay Free Recall** | **rs = .629, p=.021** | n.s. | n.s. | n.s. | n.s. | n.s. | **rs = -.591, p=.033** |
| **Long Delay Rotated Recall** | **rs = .559, p=.047** | n.s. | n.s. | n.s. | n.s. | n.s. | **rs = -.621, p=.023** |
| **Recognition Hits** | **rs = .620, p=.024** | n.s. | n.s. | n.s. | n.s. | ***rs = -.787, p=.001*** | *rs = -.480, p=.097* |
| Recognition False Positives | n.s. | n.s. | n.s. | n.s. | n.s. | n.s. | *rs = .516, p=.071* |
| Loss after consolidation | n.s. | n.s. | n.s. | n.s. | n.s. | n.s. | n.s. |
| Proactive Interference | n.s. | n.s. | n.s. | n.s. | n.s. | n.s. | n.s. |
| Retroactive Interference | n.s. | n.s. | n.s. | n.s. | n.s. | n.s. | n.s. |

No correlation survived Bonferroni’s correction for multiple comparison. p<.05 in **bold**, p<.1 in *italics*.
